# Alcohol abstinence stigma and alcohol use among HIV patients in Thai Nguyen, Vietnam

**DOI:** 10.1371/journal.pone.0239330

**Published:** 2020-09-30

**Authors:** Kathryn E. Lancaster, Angela Hetrick, Teerada Sripaipan, Tran Viet Ha, Heidi E. Hutton, Geetanjali Chander, Carl A. Latkin, David Dowdy, Constantine Frangakis, Bui Xuan Quynh, Vivian F. Go

**Affiliations:** 1 Division of Epidemiology, College of Public Health, The Ohio State University, Columbus, OH, United States of America; 2 Department of Health Behavior, Gillings School of Global Public Health, University of North Carolina at Chapel Hill, Chapel Hill, NC, United States of America; 3 Department of Psychiatry and Behavioral Sciences, Johns Hopkins School of Medicine, Baltimore, Maryland, United States of America; 4 Department of Epidemiology, Johns Hopkins Bloomberg School of Public Health, Baltimore, Maryland, United States of America; 5 General Internal Medicine, Johns Hopkins School of Medicine, Baltimore, Maryland, United States of America; 6 Department of Health, Behavior and Society, Johns Hopkins Bloomberg School of Public Health, Baltimore, Maryland, United States of America; 7 Department of Biostatistics, Johns Hopkins Bloomberg School of Public Health, Baltimore, Maryland, United States of America; 8 The University of North Carolina Project in Vietnam, Hanoi, Vietnam; University of the Witwatersrand, SOUTH AFRICA

## Abstract

**Background:**

Hazardous alcohol use is prevalent among people living with HIV (PWH), leading to sub-optimal HIV treatment outcomes. In Vietnam, alcohol use is highly normative making it socially challenging for PWH to reduce or abstain. We used mixed methods to develop a quantitative scale to assess alcohol abstinence stigma and examined the association between alcohol abstinence stigma with alcohol use among PWH in Vietnam.

**Methods:**

We conducted qualitative interviews with 30 PWH with hazardous alcohol use from an antiretroviral therapy (ART) clinic in the Thai Nguyen to inform item development. Alcohol use was assessed using the Alcohol Use Disorders Identification Test. We tested items in a survey of 1,559 ART clinic patients to assess internal consistency and structural validity. We used log binomial modeling to estimate associations between any reported alcohol abstinence stigma and alcohol use.

**Results:**

Using the results from the qualitative interview data, we developed the alcohol abstinence stigma scale with seven final items with scores ranging from 0 (no stigma) to 28 (high stigma). The scale had good internal consistency (α = 0.75). Exploratory factor analysis suggested the presence of three factors: internalized, experienced, and anticipated stigma that explained 56.9% of the total variance. The mean score was 2.74, (SD = 4.28) and 46% reported any alcohol abstinence stigma. We observed a dose-response relationship between alcohol abstinence stigma and alcohol use. PWH who reported any alcohol abstinence stigma had greater hazardous alcohol use (aPR = 1.32, 95% CI: 1.12, 1.56), harmful alcohol use (aPR = 2.26, 95% CI: 1.37, 3.72), and dependence symptoms (aPR = 3.81, 95% CI: 2.19, 6.64).

**Conclusion:**

Alcohol abstinence stigma is associated with increased alcohol levels of alcohol use among PWH in Vietnam, signaling challenges for alcohol reduction. Consideration of alcohol abstinence stigma will be essential for the design of effective alcohol reduction interventions and policy efforts to prevent adverse health consequences of alcohol use among PWH.

## Introduction

Alcohol use and HIV are intrinsically intertwined with increased risk for adverse health outcomes. Persons that engage in heavy alcohol use are at an increased risk for HIV acquisition through multiple sexual partnerships, unprotected sex, and injection drug use [1, 2]. People living with HIV (PWH) who use alcohol are twice as likely to be non-adherent to their ART treatment [3, 4]. These behaviors are correlated with reduced viral suppression and contribute to an increase in forward transmission of HIV [5]. PWH may be motivated to reduce alcohol consumption in accordance with their antiretroviral therapy (ART) regimen, per physician recommendations [6]. However, alcohol reduction may be challenging in contexts where alcohol use is highly pervasive and serves as a social lubricant [7–9].

Alcohol use disorders remain prevalent in Vietnam and can lead to alcohol-related adverse health outcomes such as chronic disease, HIV acquisition, traffic-related hospital admissions, fatalities, and overall disease burden [1, 10–13]. Alcohol is a leading cause of years of life (YLL) lost in Vietnam; in 2016, WHO classified Vietnam within the second-highest quintile of alcohol-attributable YLL among all nations [11, 14]. Compared to the general Vietnamese population and their HIV positive peers, PWH who consume alcohol hazardously report a lower health-related quality of life [3, 15].

Cultural and gender norms may lead to prevalent and normative alcohol use in many settings, including Vietnam [6, 7, 12]. Alcohol use disorders (AUD) are common among men in Vietnam, such as substantial prevalence in rural districts (26.2%) and male university students engaging in alcohol-related harms (81.8%) [16–18]. Alcohol consumption is perceived as tantamount to masculinity and the social pressure to drink heavily is high [19]. For men, consuming alcohol with business colleagues and superiors is normative and a requirement for many professions [12]. It is often socially and professionally damaging for Vietnamese men to decline alcohol. The pressure to consume shots of liquor in unison and the phrases such as "not drunk, not going home," ensure intoxication among all present [12, 19]. While men are expected to drink heavily and remain high-functioning, women are expected to drink less; however, rates of alcohol consumption among women are increasing which will likely impact current gender norms [6, 12]. Although the high proportion of men in Vietnam living with AUD is well-known, the percentage of women with AUD is likely severely underestimated due to experienced stigma from gender norms and social pressure to conceal alcohol use [20].

The social context surrounding alcohol use may inhibit PWH's ability to reduce or abstain from alcohol use. Societal norms of heavy alcohol use and internalized and experienced stigma make abstaining or reducing alcohol consumption challenging for PWH in Vietnam [6]. For example, abstaining from alcohol may indicate to peers that a person is living with HIV; therefore, PWH, especially those who are socially isolated, feel increased social pressure to drink to avoid disclosing their HIV status [6]. This potential dual stigma- HIV and alcohol abstinence- has not yet been addressed for PWH. Although stigma is generally associated with harmful health behaviors such as injection drug use or sex work, safe health behaviors may be stigmatized [21–23]. Currently, there are no quantitative measures to capture alcohol abstinence stigma.

To develop interventions that reduce heavy alcohol use and HIV transmission, it is vital to assess the potential stigma associated with alcohol use in a society where alcohol use is a social and professional lubricant. In this study, we developed an alcohol abstinence stigma scale that quantifies alcohol abstinence stigma among PWH in Thai Nguyen, Vietnam. Given the potential for stigma to inhibit one's ability to reduce or abstain from alcohol use, we examined the relationship between alcohol abstinence stigma and alcohol use disorders among this population.

## Methods

### Study location

Study participants were recruited from the small-urban province of Thai Nguyen in North Vietnam, about 80 km outside of the capital city Hanoi. Of the twelve government clinics within Thai Nguyen, the seven ART clinics with the highest number of ART clients were selected to recruit participants.

### Scale development

We completed a qualitative analysis as an initial phase of the Reducing hazardous alcohol use & HIV viral load: an RCT in ART Clinics in Vietnam (REDART) study. The REDART study is a three-arm randomized control trial (RCT) among hazardous and heavy alcohol drinking HIV-infected ART clinic patients in Thai Nguyen, Vietnam. To inform and develop a measurement of a stigma when abstaining from alcohol included in the core REDART survey, we conducted in-depth interviews with 30 ART clinic patients, performed literature reviews, and consulted with content experts.

Participants were required to meet the following eligibility criteria, 1) 18–64 years of age, 2) currently receiving ART, 3) attained an 8 or higher on the Alcohol Use Disorder Identification Test (AUDIT), and 4) resided in the Thai Nguyen province. ART clinic providers introduced the study to patients and referred interested patients to a private room within the clinic for more information. Trained recruiters provided study information, obtained verbal consent in Vietnamese, and administered the AUDIT to prospective participants [24]. The AUDIT has demonstrated validity and reliability in diagnosing alcohol dependence in rural Vietnamese communities [17]. Participants were classified as high risk of alcohol dependence using the recommended AUDIT cutoff score of 8.

Interviews were transcribed and translated word for word. Each transcript was independently coded by two reviewers using a codebook with conceptual attributes identified in a literature review. We developed a final codebook integrating the interview guide, interview summaries, and common themes and characteristics that emerged from a review of the transcripts. Codes and definitions were revised until emerging themes were exhausted. We used thematic and content methods to analyse the qualitative data [25]. QRS NVivo11 was used to manage data and code transcripts. Based on the qualitative analysis, literature review, and content feedback, we developed items representing three dimensions of alcohol abstinence stigma, including internalized, experienced, and anticipated stigma (Table 1) [26]. Within the qualitative interviews, participants revealed feelings of shame or embarrassment that came with refusing drinks. Many also noted that they were concerned about facing isolation, mocking, or ridicule if they abstained or reduced their alcohol use. Several participants also described the expectations to drink at celebrations or work functions. The scale items were grounded in the themes which emerged from the qualitative data.

**Table 1:** Exemplary quotes of internalized, experienced, and anticipated stigma from ART clinic patients in Vietnam (n = 30) with associated domains and alcohol abstinence stigma scale items.

	Exemplary Quotes	Survey Item
*Internalized Stigma*		
	Fellows give a toast. If I cannot drink I feel ashamed	I feel ashamed when I decline to drink.
	Q: Did you feel uneasy for not drinking with friends?	I feel embarrassed when I decline to drink.
	A: Yes, I did.	
*Experienced Stigma*		
	If I quit drinking successfully, I would just stay alone!	I become isolated from my family when I do not drink.
	It means, if friends offer alcohol to you, but you don't join, they may think that you disregard them	
	It makes friends feel closer to one another. Inviting friends to go out and drink once in a while will make us feel closer to each other, and we will find it easier to say the things that we normally cannot.	I become isolated from my friends when I do not drink.
*Anticipated Stigma*		
	I’m afraid that I cannot quit as there are banquets or anniversaries in which drinking is a part	I feel forced to drink at celebrations, such as a wedding or funerals
	When men from other table say that they want to toast for our health (a group of women), how can we refuse?	
	You see, when I have guests or when I am invited by my friends, I drink li\le. I mainly drink on my family’s occasions. For instance, at the weddings of my kindred families, I drink much because my cousins, my aunts, my uncles and my descendants want me to drink with them.	
	Yes, mocking (laughing), such as: “Are you afraid of your wife” or “do you feel sorry to spend money on this” … they try to find any reason to force their friends drinking.	I am mocked when I do not drink.
	To be frank, if I totally stop drinking I have to do the following. If I could stop drinking from now on, I have to stop working, will not meet and exchange with friends and I just do family work not drinking “prevents my work”	My business relationships will suffer if I stop drinking.

Cognitive interviews were conducted with 5 ART clinic patients to confirm the acceptability of the final scale items for comprehension and cultural appropriateness. Within 24 hours of each cognitive interview, the interviewer wrote detailed summary notes and debriefed with the rest of the research team to review problematic areas and made revisions as appropriate. The qualitative analysis, literature review, data collection, and cognitive interviews resulted in a final alcohol abstinence stigma scale that consisted of 7 items that focused on internalized, anticipated, and experienced stigma (S1 Fig). Participants preferred to respond to each item using a 10-point Likert scale from 'Strongly Disagree' (value of 1) to 'Strongly Agree' (value of 10).

### Survey implementation

A descriptive cross-sectional analysis was conducted using baseline survey data from REDART, our ongoing alcohol reduction randomized control trial. Study interviewers introduced the REDART project to each interested ART client. At the time of screening, those aged 18 years or older and interested in the study were administered a baseline written informed consent. The target study population were ART clinic patients in Thai Nguyen who met the minimum t-cell count (CD4^+^>350 cells/mm^3^) requirements to receive ART under Vietnam's Ministry of Health 2013 guidelines. Study eligibility also required participants to reside in Thai Nguyen for 24 months following study enrollment. Surveys were administered from March 28, 2016 through May 19, 2017.

Baseline surveys were administered by trained, non-ART clinic research staff who used tablets to collect data. Participants completed the survey in a private location within their enrolled ART clinic. The approximate length of the survey was 1–2 hours and consisted of baseline risk assessments such as AUDIT and other alcohol use items, drug use, sexual risk behaviors, injecting risk behaviors, sociodemographic information, and self-reported adherence to ART. Participants were compensated with 100,000 Vietnamese Dong [US $5.00] and travel reimbursement.

### Scale validation

The internal consistency and reliability of the developed scale were measured by calculating Cronbach's alpha. To determine the underlying constructs within the scale, we conducted an exploratory factor analysis (EFA) with an iterated principal factor analysis method to account for non-normal distribution of item responses and an oblique rotation to account for correlation between scale items. A Scree test, parallel analysis, and root mean squared error of approximation methods were conducted to determine the number of common factors to be included in the analysis. Each stratification of alcohol use was compared using the Wilcoxon two-sample test to compare the alcohol abstinence score means and for each scale item.

### Statistical analyses

Our analyses consisted of three dependent variables based on the classification of types of alcohol use: hazardous, harmful, and alcohol dependence. Classifications of types of alcohol use were determined by the Alcohol Use Disorders Identification Test (AUDIT). Developed by the World Health Organization (WHO), the AUDIT helps practitioners screen persons for excessive drinking through three domains: hazardous alcohol use, alcohol dependence, and harmful alcohol use [27].

We used the optimal screening thresholds for hazardous alcohol use as cutoff points [24]. ART clinic patients were classified as ‘hazardous alcohol users’ if their total AUDIT score was between 8 and 15, ‘harmful alcohol users’ if they scored 16 to 19, and as ‘alcohol dependent users’ if their total score was 20 or higher [28]. ART clinic patients who did not answer all seven alcohol abstinence questions (n = 16) and those who did not answer all AUDIT questions (n = 189) were excluded from the study.

We calculated frequency distributions for categorical variables and medians and inter-quartile ranges for continuous variables. The primary outcome measures were binary variables: hazardous alcohol use, harmful alcohol use, and alcohol dependence symptoms as predicted by the primary binary exposure variable of alcohol abstinence stigma. The scale was condensed to a 5-point Likert scale to adjust for a right-skewed distribution, likely due to alcohol use as a societal norm, through an equalization method; values of 1 and 2 were combined and recoded to represent a score of ‘0’, and this pair process was repeated through values 9 and 10 representing a score of ‘4’. Reported alcohol abstinence stigma was classified as ‘1’for any score greater than a score of 0, and ‘0’ for no reported alcohol abstinence stigma. The scale headings for each stigma item ranges from ‘Strongly Disagree’ with a value of ‘0’, to ‘Neither Disagree or Agree’ with a value of ‘2’ and ‘Strongly Agree’ with a value of ‘4’ (S1 Fig). Based on this scale structure and the total values ranging from 0 to 28, we hypothesize that equally distributing the values between low (0–8) and moderate to high (9–28) levels of stigma will best classify the level of alcohol abstinence stigma felt by participants.

A review of peer-reviewed literature informed a set of a prior confounders to include in our analysis of the association between alcohol abstinence stigma and alcohol use [16–18, 29–31]. Covariates examined included: age (continuous variable), gender (male, female), education (completion of secondary school), relationship (currently married), employment (employed full or part-time), and homelessness in the past three months (no, yes). Covariates were categorized to support interpretability and replicability of the study results.

We used log-binomial modeling to estimate unadjusted prevalence ratios and 95% confidence intervals for the dependent variables; hazardous alcohol use, harmful alcohol use, and alcohol dependence [32]. Potential confounding variables were assessed using a univariate test with the outcome (α < 0.10). Confounding variables were kept in the final adjusted multivariable model if their absence changed the prevalence ratio estimate by more than 10% [33, 34]. The data analysis for this paper was generated using SAS 9.4 software of SAS Institute Inc., Cary, NC, USA.

The study was approved by the University of North Carolina Chapel Hill and Thai Nguyen Center for Preventive Medicine Institutional Review Boards.

## Results

### Study population characteristics

Baseline survey data were collected on 1,362 HIV-infected ART clinic patients among Thai Nguyen Province ART clinics (Table 2). Most participants were male (77.6%) and the overall median age was 39 years (IQR = 35–43). Approximately one-sixth of participants (15.7%) had completed high school or above, and 65.3% were currently married or living with a partner.

**Table 2:** Characteristics of ART clinic patients in Thai Nguyen, Vietnam.

	Total study population	Non-Hazardous Alcohol Use	Hazardous Alcohol Use	Harmful Alcohol Use	Alcohol Dependence
	(N = 1362)	(n = 838; 61.6%)	(n = 380; 27.9%)	(n = 70; 5.1%)	(n = 74; 5.4%)
Baseline characteristics	*n* (%)	*n* (%)	*n* (%)	*n* (%)	*n* (%)
Sex					
Male	1057 (77.6)	549 (65.5)	366 (96.3)	69 (98.6)	73 (98.7)
Female	305 (22.4)	289 (34.5)	14 (3.7)	1 (1.4)	1 (1.3)
Age^a^					
Median (IQR)	39 (35–43)	39 (35–43)	39 (35–43)	39 (35–43)	39 (35–43)
Homeless past 3 months					
Yes	13 (1.0)	6 (0.7)	3 (0.8)	1 (1.4)	3 (4.1)
No	1349 (99.0)	832 (99.3)	377 (99.2)	69 (98.6)	71 (95.9)
Education					
High school or more	214 (15.7)	179 (21.4)	83 (21.8)	11 (15.7)	14 (18.9)
No high school	1148 (84.3)	659 (78.6)	297 (78.2)	59 (84.3)	60 (81.1)
Relationship Status					
Not married or living with partner	473 (34.7)	345 (41.2)	117 (30.8)	18 (25.7)	29 (39.2)
Married or living with partner	889 (65.3)	493 (58.8)	263 (69.2)	52 (74.3)	45 (60.8)
Employment Status					
full time	682 (50.1)	402 (48.0)	214 (56.3)	39 (55.7)	27 (36.5)
less than full time	680 (49.9)	436 (52.0)	166 (43.7)	31 (44.3)	47 (63.5)

^a^Missing 43 responses due to not knowing or refused to answer.

Alcohol use disorders within the AUDIT are ordered in increasing severity of alcohol use as hazardous alcohol use, harmful alcohol use, and alcohol-dependent symptoms [27]. Approximately one-fourth of the study population were classified as hazardous alcohol users (27.9%), and a minority met criteria for harmful alcohol use (5.1%) or exhibited alcohol dependence symptoms (5.4%).

### Scale reliability and validity

We identified 3 factors using the Scree test and 4 factors using the upper bound of a parallel analysis. The RSMEA method using the maximum likelihood estimate produced a poor fit for 2 factors (RMSEA = 0.17) and a close fit for 3 factors (RMSEA = 0.04). Based on these results, we used three common factors in the exploratory factor analysis. Communalities of factors ranged from 0.26 to 0.80.

The first factor, internalized stigma, consisted of two items and accounted for 36.8% of the total variance. Factor loadings were high (0.85, 0.88) for both items: ‘I feel ashamed when I decline to drink’ and ‘I feel embarrassed when I decline to drink.’

The second factor, experienced stigma, consisted of two items and accounted for 11.3% of the total variance. Factor loadings were high (0.63, 0.86) for both items: ‘I become isolated from my family when I do not drink’ and ‘I become isolated from my friends when I do not drink.’

The third factor, anticipated stigma, consisted of three items and accounted for 8.8% of the total variance. Factor loadings were adequate to high (0.56, 0.80, 0.33) for the following items: ‘I am mocked when I do not drink,’ ‘I feel forced to drink at celebrations, such as weddings or birthdays,’ and ‘my business relationships will suffer if I stop drinking.’

The developed scale was internally consistent (Cronbach’s α = 0.75) for participants (n = 1362) who answered all seven stigma questions and all AUDIT question items. Internal consistency was adequate within all three factor subscales (Cronbach’s α = 0.86, 0.69, 0.64, respectively).

### Scale scoring

Wilcoxon two-sample testing calculated significant differences in the overall survey mean and survey item means between each alcohol use disorder classification and the referent group, non-hazardous alcohol users (Table 3). Each participant can score from no stigma (score = 0) to the highest possible level of stigma experienced (score = 28). The mean score for all participants was low (2.74, (SD = 4.28)). Those with symptoms of alcohol dependence experienced the most alcohol abstinence stigma (mean = 6.70, SD = 4.92). Non-hazardous alcohol users experienced the least amount of alcohol abstinence stigma (mean = 2.35, SD = 3.96). There were significant differences in total score and for most scale items between those with and without each classification of an alcohol use disorder.

**Table 3:** Comparison of alcohol abstinence stigma scale mean scores among ART clinic patients in Thai Nguyen, Vietnam.

*Scale Item*	Total Study Population	Non-Hazardous Alcohol Use	Hazardous Alcohol Use	Harmful Alcohol Use	Alcohol Dependence
	(n = 1362)	(n = 838)	(n = 380)	(n = 70)	(n = 74)
	(SD)	(SD)	(SD)	(SD)	(SD)
*Total Score (out of 28)*	2.74	1.70	3.81**	5.19**	6.70**
	(4.28)	(3.19)	(5.04)	(5.46)	(4.92)
*I feel ashamed when I decline to drink*.	0.48	0.26	0.71**	0.93**	1.42**
	(1.10)	(0.84)	(1.28)	(1.32)	(1.55)
*I feel embarrassed when I decline to drink*.	0.41	0.22	0.59**	0.81**	1.28**
	(1.02)	(0.77)	(1.19)	(1.31)	(1.51)
*I become isolated from my family when I do not drink*.	0.07	0.04	0.12**	0.17**	0.11**
	(0.42)	(0.31)	(0.54)	(0.68)	(0.42)
*I become isolated from my friends when I do not drink*.	0.13	0.10	0.16	0.24*	0.30**
	(0.57)	(0.50)	(0.65)	(0.77)	(0.74)
*I am mocked when I do not drink*.	0.51	0.31	0.73**	1.00**	1.16**
	(1.09)	(0.83)	(1.29)	(1.46)	(1.49)
*I feel forced to drink at celebrations such as birthdays or weddings*.	0.79	0.58	1.01**	1.20**	1.61**
	(1.29	(1.09)	(1.46)	(1.46)	(1.65)
*My business relationships will suffer if I stop drinking*	0.34	0.19	0.49**	0.83**	0.82**
	(0.93)	(0.68)	(1.09)	(1.41)	(1.37)

*One-sided Wilcoxon two-sample test <0.05 with ‘yes’ as referent group.

**One-sided Wilcoxon two-sample test <0.01 with ‘yes’ as referent group.

### Convergent validity: Associations with alcohol use

A dose-response relationship was observed between any alcohol abstinence stigma and increased alcohol use (Table 4). Overall, 46% (n = 625) reported any alcohol abstinence stigma. In multivariable analysis adjusting for gender, PWH who reported alcohol abstinence stigma were more likely to be hazardous drinkers (adjusted PR: 1.52, 95% CI: 1.30, 1.79) or harmful drinkers (adjusted PR: 2.96, 95% CI: 1.82, 4.82) when compared to those who did not report alcohol abstinence stigma. PWH who reported alcohol abstinence stigma were 4.74 (95% CI: 2.75, 8.19) times as likely to report alcohol-dependent symptoms compared to those who did not report alcohol abstinence stigma, adjusting for gender.

**Table 4:** Associations of alcohol abstinence stigma and alcohol use.

	Hazardous Alcohol Use		Harmful Alcohol Use		Alcohol Dependence	
	PR (%)	aPR^a^	PR (%)	aPR^a^ (%)	PR (%)	aPR^a^ (%)
	(95% CI)	(95% CI)	95% CI	95% CI	95% CI	95% CI
No Alcohol Abstinence Stigma	1.00	1.00	1.00	1.00	1.00	1.00
Alcohol Abstinence Stigma	1.84	1.52	3.77	2.96	6.11	4.74
	(1.56, 2.18)	(1.30, 1.79)	(2.30, 6.18)	(1.82, 4.82)	(3.52, 10.61)	(2.75, 8.19)

PR = prevalence ratio; CI = confidence interval; aPR = adjusted prevalence ratio.

^a^Adjusted for gender.

## Discussion

Developing a measure of alcohol abstinence stigma is essential for the design of effective alcohol reduction interventions and policy efforts to prevent adverse health consequences of alcohol use among PWH. Stigma is often associated with harmful health behaviors, yet safe behaviors, such as abstaining from alcohol, may be stigmatizing [21–23]. We developed a seven-item alcohol abstinence stigma scale that can be used to determine the presence of internalized, anticipated, and experienced stigma among PWH in Thai Nguyen, Vietnam. This scale performed well in factor analyses and demonstrated adequate reliability. Alcohol abstinence stigma should be evaluated among PWH in settings where alcohol use is culturally encouraged.

Overall, the prevalence of reported alcohol abstinence stigma was low among our sample of ART patients in Thai Nguyen, Vietnam. Our qualitative work among PWH in Thai Nguyen identified a strong desire among participants to reduce their alcohol consumption. However, participants also expressed difficulty in reducing their drinking due to sigma related to abstaining from alcohol in social, family, and work settings [6]. Further, our results demonstrated a dose-response relationship between alcohol abstinence stigma and alcohol use disorder severity. Our findings align with previous qualitative findings that PWH in Thai Nguyen desire to reduce their alcohol consumption, but find it difficult due to stigma related to abstaining from alcohol in social, family, and work settings [6]. Most notably, PWH stated, “Drinking is like a rule you can’t break [6]” in regards to social settings in Vietnam.

We found that alcohol abstinence stigma was associated with alcohol use, including hazardous, harmful, and alcohol-dependent drinking. Alcohol use is seen as a critical component in social settings, prompting PWH in Vietnam to express internalized and experienced stigma when attempting to reduce alcohol consumption [6]. The low prevalence of alcohol abstinence stigma among participants paired with our definition of alcohol abstinence stigma warrant cautious interpretation of the results. We interpreted our scale to measure any reported stigma against the lowest potential value of stigma that a participant could report. Additionally, due to alcohol use norms in Vietnam, a condensed 5-item Likert response may be appropriate to account for a skewed distribution. More work is needed to test this scale and responses with PWH in similar settings who live in areas where alcohol use is a common societal norm to determine if higher values of alcohol abstinence stigma are reported.

Future alcohol reduction interventions need to explore opportunities for new social support groups for non-drinkers to address social pressures and potential alcohol reduction stigma. Caution, however, must be used in drawing causal and temporal interpretations of this cross-sectional analysis, particularly in concluding alcohol abstinence stigma may predict alcohol use. For example, those with non-hazardous drinking may be less likely to report alcohol abstinence stigma because their drinking patterns may be less stigmatized. Nonetheless, further longitudinal evaluations are needed to untangle how alcohol abstinence stigma may influence alcohol use patterns and reduction efforts among PWH.

The internal consistency for our scale was not high for all items included in the scale, likely due to our factors only including two to three items. Nonetheless, completion of the EFA resulted in the improvement of internal consistency by moving two items from the ‘experienced’ to ‘anticipated’ category, ‘I am mocked when I do not drink’ and ‘I feel forced to drink at celebrations such a wedding or funerals.’ Additionally, EFA strongly supported the three factors of stigma: internalized, experienced, and anticipated. This is likely the product of our development approach, in which factors of stigma and the corresponding items were determined using qualitative analysis, literature review, data collection and cognitive interviews. Future studies that use confirmatory factor analysis on a larger, validation sample will be useful to validate the findings of the EFA further.

It is important to note that all the measures included in this study were self-reported. Social desirability may have biased effects of the associations between alcohol abstinence stigma and alcohol use towards a null association in two ways. First, social desirability to report consuming a higher amount of alcohol may have misclassified those who were not heavy alcohol users as reporting heavy alcohol use. Second, social desirability for high alcohol consumption to be perceived as manageable and socially acceptable may have prompted low abstinence stigma scores overall. Items within the AUDIT have also led to false positives among heavy drinkers [35], which could further lead to a null association between alcohol abstinence stigma and alcohol consumption. To minimize recall and social desirability bias, we conducted face-to-face interviews with interviewers who were trained extensively in non-judgmental interviewing techniques.

Despite being an important social and cultural construct that may inhibit alcohol reduction strategies, alcohol abstinence stigma has not been quantitatively measured to our knowledge. This scale was tested among a limited sample of HIV patients in Vietnam. Findings may differ in other contexts and warrant further evaluations in other settings where alcohol is highly normative. The scale should also be implemented among PWH and people living without HIV to determine if those living with HIV experience more alcohol abstinence stigma. In Vietnam specifically, approximately 19.8% of adults in urban and rural areas are hazardous or harmful alcohol drinkers [7], compared with 30% of PWH [2]. This new measure will aid future studies to assess the value of developing culturally sensitive strategies to reduce alcohol consumption and ultimately improving HIV treatment outcomes among PWH.

## Conclusion

We developed a reliable scale to measure alcohol abstinence stigma experienced among people living with HIV in Thai Nguyen, Vietnam. PWH who reported alcohol abstinence stigma were more likely to be hazardous, harmful, or alcohol-dependent drinkers than those who did not report alcohol abstinence stigma. Assessing alcohol abstinence stigma may be a critical element for the design of effective alcohol reduction interventions and policy efforts to reduce alcohol consumption for PWH. Alcohol reduction efforts should explore the development of new social support programs to consider social pressures and potential stigma to reducing alcohol use.

## Supporting information

S1 Fig Alcohol abstinence stigma scale survey tool. (DOCX)
