# Supplementary material for: Alcohol abstinence stigma and alcohol use among HIV patients in Thai Nguyen, Vietnam
Source: PLoS One. 2020 Sep 30;15(9):e0239330. doi: 10.1371/journal.pone.0239330 (PMC7526924; doi:10.1371/journal.pone.0239330)
Supplement: S1 Fig — (DOCX) [file pone.0239330.s001.docx]

S1 Fig. Alcohol Abstinence Stigma Scale Survey Tool

| *[Interviewer: move across rows for response options]* | **0**  **Strongly disagree** | **1** | **2**  **Neither disagree nor agree** | **3** | **4**  **Strongly agree** | **[Don’t read]**  **Refuse** | **[Don’t read]**  **DK** |
| --- | --- | --- | --- | --- | --- | --- | --- |
| 1. I feel ashamed when I decline to drink.   [FOR TRANSLATION: The word “ashamed” is meant to feel guilty or regretful] | _(0)_ | _(1)_ | _(2)_ | _(3)_ | _(4)_ | _(88)_ | _(99)_ |
| 1. I feel embarrassed when I decline to drink.   [FOR TRANSLATION: The word “embarrassed” is meant to feel awkward or self-conscious] | _(0)_ | _(1)_ | _(2)_ | _(3)_ | _(4)_ | _(88)_ | _(99)_ |
| 1. I become isolated from my family when I do not drink. | _(0)_ | _(1)_ | _(2)_ | _(3)_ | _(4)_ | _(88)_ | _(99)_ |
| 1. I become isolated from my friends when I do not drink. | _(0)_ | _(1)_ | _(2)_ | _(3)_ | _(4)_ | _(88)_ | _(99)_ |
| 1. I am mocked when I do not drink. | _(0)_ | _(1)_ | _(2)_ | _(3)_ | _(4)_ | _(88)_ | _(99)_ |
| 1. I feel forced to drink at celebrations, such as a wedding or birthdays. | _(0)_ | _(1)_ | _(2)_ | _(3)_ | _(4)_ | _(88)_ | _(99)_ |
| 1. My business relationships will suffer if I stop drinking | _(0)_ | _(1)_ | _(2)_ | _(3)_ | _(4)_ | _(88)_ | _(99)_ |
